# Function of SYDE C2-RhoGAP family as signaling hubs for neuronal development deduced by computational analysis

**DOI:** 10.1038/s41598-022-08147-7

**Published:** 2022-03-12

**Authors:** Zen Kouchi, Masaki Kojima

**Affiliations:** 1 Department of Genetics, Institute for Developmental Research, Aichi Developmental Disability Center, 713-8 Kamiya-cho, Kasugai, Aichi 480-0392 Japan; 2 Laboratory of Bioinformatics, School of Life Sciences, Tokyo University of Pharmacy and Life Sciences, 1432-1 Horinouchi, Hachioji, 192-0392 Japan

**Keywords:** Bioinformatics, Cell signalling, Molecular modelling

## Abstract

Recent investigations of neurological developmental disorders have revealed the Rho-family modulators such as Syde and its interactors as the candidate genes. Although the mammalian Syde proteins are reported to possess GTPase-accelerating activity for RhoA-family proteins, diverse species-specific substrate selectivities and binding partners have been described, presumably based on their evolutionary variance in the molecular organization. A comprehensive in silico analysis of Syde family proteins was performed to elucidate their molecular functions and neurodevelopmental networks. Predicted structural modeling of the RhoGAP domain may account for the molecular constraints to substrate specificity among Rho-family proteins. Deducing conserved binding motifs can extend the Syde interaction network and highlight diverse but Syde isoform-specific signaling pathways in neuronal homeostasis, differentiation, and synaptic plasticity from novel aspects of post-translational modification and proteolysis.

## Introduction

Numerous adhesion molecules regulate synapse development by recruiting presynaptic and postsynaptic components to the synaptic clefts. Several RhoGTPase-activating proteins (RhoGAPs) have been implicated as critical regulators of these processes. Synapse-defective-1 protein (Syde1) is an essential regulator of the presynaptic developmental organization. It’s role was demonstrated by the loss of synaptic recruitment and neuronal pathfinding in genetic studies on Syd1, Syde1 ortholog mutants in *C. elegans* and *Drosophila*^1,2^. Scheiffele’s group also demonstrated a similar function for Syde1 in vertebrates by identifying abnormal synaptic clustering in knockout mice brains and dissociated neuronal cultures^3^. The N-terminal disordered region of mouse Syde1 is important for the synaptogenic activity and the RhoGAP domain regulates dendritogenesis. The RhoGAP activity of *Drosophila* Syd1 regulates the clustering of Bruchpilot (Brp) as the active zone component^3,4^, suggesting that the domain function and regulation differ among the species. Although mouse and *Drosophila* Syde1 possess RhoGAP activity^3,4^, no GAP activity has been detected in worm Syde1, which binds to GTP-bound MIG-2/Rac^4,5^. It has been suggested that SYDE1 is conserved phylogenetically among worms, *Drosophila*, and mice. Yet, given the molecular organization, the activation of Syde1 by upstream signaling, and the overlap between the functions of Syde2 and Syde1 in mammalian development are unclear^2,3^. Another knockout study of Syde1 in mice showed that it regulates trophoblast migration and invasion during the development of placentas by remodeling the cytoskeleton; it is also involved in the fetal growth regulation during gestation^6^. Regulation of the Syde1 expression by the transcriptional factor glial cell missing 1 (GCM1) during placentation suggests a mammalian-specific developmental signaling cascade that contrasts with the impairment of synaptic vesicle docking commonly observed among their mutant organisms^3,4,7^.

Mouse Syde1 forms a complex with Munc18-1 and liprin-α2 through the N-terminal disordered domain, the presynaptic organizers necessary for synaptogenesis. The Syde1 binding domain in liprin-α2 mediates the interaction with CASK^3,8^. Liprin-α2 organizes type IIa LAR-RPTP complex through selective liprin-α isoforms, implicating that Syde1 is subjected to complex regulation in the presynaptic compartment by the neuronal adhesion machinery^9^. Transcriptomic meta-analysis revealed Syde1 as one of the differentially expressed genes in the cortex of autistic patients^10^. A clinical study indicated that Syde2 is also a causative gene for intellectual disability. Nonsense mutations in Syde2 that theoretically generate a C-terminal- truncated protein devoid of the RhoGAP domain might cause its degradation, and dysregulation of axonal guidance^11^; the neuronal function of mammalian Syde2 is unknown. Pleckstrin homology domain-containing family G member 3 (PLEKHG3) possesses a guanidine nucleotide exchange activity and was identified as a Syde2 complex by high-throughput immunoaffinity screening^12^. Furthermore, PLEKHG3, a brain-enriched RhoGEF, is a potential candidate genes for autism^13^.

Syde-family proteins are characterized by the presence of RhoGAP and C2 motif in domain organization. The regions outside the domains have diversity in their sequences, defined mainly by disordered domains that are poorly characterized^14^. The N-terminal region of Syde1 mediates autoinhibition of the RhoGAP activity. However, the mechanism of integration of the Syde network in the cell-surface molecular architecture remains obscure^3,12^. Promiscuity of substrate specificity and the complex regulation of RhoGAP family proteins have been implicated in several studies using cell-based expression systems^6,12,15^.

The structure–function relationship of the SYDE-family proteins was elucidated by in silico modeling of structural domain by combining sequence and phylogeny. Additionally, the regulatory regions of the Syde proteins were identified based on the conserved candidate short linear motifs (SLiMs) located in the disordered domains of soluble proteins. The SLiMs provide regulatory flexible interface prerequisites for the dynamic assembly of protein complexes. Interestingly, predicted interactors highlight the mammalian Syde network involved in neuronal homeostasis, with post-translational modification and RhoGAP selectivity as synaptic scaffold molecules. The hypothesis of Syde function as a neuronal signaling hub will be worth investigating experimentally and clinically in the future.

## Methods

Sequence feature analysis. Syde1 and Syde2 (UniProt accession No. Q6ZW31 and Q5VT97, respectively) were downloaded from UniProt and NCBI Gene database. Each protein was aligned using the MAFFT multiple sequence alignment software^16^ and visualized with JalView^17^. Domain and sequence features were predicted by using InterproScan^18^ and secondary structure was predicted with PSIPRED^19^. Regions outside the predicted domains were assessed for disordered domain including short linear motifs (SLiMs) by MobiDB^20^, ELM^21^, and D_2_P_2_^22^ (Supplementary Table S1). SLiMs play critical roles in many biological processes and we predicted SLiMs localized within the disordered domains that are commonly mapped among orthologs. Since SLiMs have propensity of random occurrences^23^, binding motifs are also predicted from ANCHOR program^24^ analyzing the sequence of disordered region and energy for molecular interaction. Phosphorylation sites and domain organization were also confirmed by Phosphosite in D2P2 and ScanProsite^25^. S-palmitoylation site was searched from SwissPalm and manually curated from PUBMED database^26,27^. Presence of nuclear export signal was examined from sequence and structural aspects by NESdb database^28^.

Phylogenetic analysis. Sequences of Syde orthologs were downloaded from UniProt database to reconstruct the phylogeny of the protein family. Seventeen Syde1 and vertebrate Syde2 sequences were retrieved and multiple alignment was conducted with MAFFT using Jalview. Phylogenetic analysis was performed with MEGA11 by Maximum Likelihood based on the JTT model + G (Gamma distributed sites) with 500 bootstrap replicates^29^.

Homology modeling. Sequences of RhoGAP and C2 domain of each SYDE proteins were submitted to the homology detection method HHpred and multiple alignment-based detection was conducted with Swiss-model search, taking into account target-template secondary structure similarity^30,31^. Models for each domain was built and their model quality was assessed and estimated with transform-restrained Rosetta^32^, I-Tasser^33^, and QMEAN^34^, respectively and all the graphic images were generated by UCSF Chimera software. Evaluation of predicted model was assessed by Procheck and ERRAT^35^ (Supplementary Table S2).

Molecular docking. Modelled Syde1 and Syde2-RhoA docking analysis were performed by SwarmDock docking program, one of the best flexible docking performing programs^36^. The ability of the programs for reproducing the RhoGTPase-RhoGAP interaction was checked by 5c2k and 5c2j for face-to-face. Optimization of inter-atom energies and side-chains of modeled Syde structures were performed by EGAD program^37^ and adjusted PDB files were subjected to the SwarmDock web server in full blind mode.

Known Syde interaction analysis and Syde interactors prediction. BioGrid^38^ and IntAct^39^ databases were used for compiling a list of experimentally analyzed Syde interactors and manually curated from the literatures (Table 1, Supplementary Table S1). The binding proteins were annotated with the domain architecture and biological processes involved in physiological aspects by retrieving from the UniProt, InterPro^18^, KEGG^40^ database. Details in the interaction were inferred from PubMed with selected keywords for neuronal function.

**Table 1:** SYDE interactors. (Upper panel) The putative domains in linear motifs predicted to mediate SYDE interaction were selected. It is assumed that if the known binding protein is the same class of protein-family or presents the domain or motif known to interact with a predicted SYDE linear motif the molecule is considered to be the putative interactor binding site (SLiM: short linear motif^23^). The consensus motifs were examined by ELM database and the selected sequence patterns localized in SYDE1 and SYDE2 are indicated according to several literatures listed in the references. (Lower panel) The SYDE1, SYDE2, and *Drosophila* Syd1 (DmSyd1) interactors are indicated by categorizing from the literatures as follows: domain architecture obtained from InterPro database, the experimental condition, and interacting region which is experimentally validated (–: not determined). *AP-MS* affinity purification-mass spectrometry, *FLAP* fluorescence recovery after photobleaching, *Y2H* yeast two-hybrid system: coIP: immunoprecipitation. *Predicted nuclear export signal is indicated by NES database^28^.

Protein	Predicted interactor	Domain architecture	Signaling pathway	Method	Score (ELM)	Predicted SYDE1 or SYDE2 interacting region
SYDE1	ATG8 (GABARAP)	ATG8, LIR domain	Autophagic pathway	ELM: LIG_LIR_Gen1^55^	3.60e−03	Y_LIR sequence ^9^TFSRL^13^
SYDE1	Ruk/CIN85	SH3 domain	Synaptic endocytosis	ELM:LIG_SH3_3 ^68^	1.32e−02	^207^DSSVGGP^213^ ^233^GDSPERP^239^ ^641^PEVVTRP^647^
SYDE1	YWHAZ	14-3-3	Neuronal differentiation	ELM: LIG_14-3-3 14-3-3 binding motif^53^	4.48e−03	Arg containing phospho-motif ^177^RRRLSLR^183^
SYDE1	Cyclin B/CDK1	cyclin	Neuronal differentiation	ELM: DOC_Cyclin_RxL S/T-X-(X)-R/K motif^54^ (peptide library)	4.21e−03	RxL docking motif ^166^RLSIKMKKLPE^175^
SYDE1	P38α, p38β	kinase	Neuronal homeostasis	ELM: DOC_MAPK Classical D motif specificity^52^	4.32e−03	^617^RPKRQPPLHL^626^
SYDE1	Casein kinase Iγ	kinase	Endocytosis synaptic vesicles	ELM: MOD_CK1_1, D_2_P_2_ Palmitoylation-dependent phosphorylation^49^	1.70e−02	^645^SPPSNRY^651^
SYDE1	Casein kinase II	kinase	Dendritic spine formation	ELM: MOD_CK2_1, D_2_P_2_, PROSITE^25^	1.46e−02	pS/pT-D/E-X-D/E motif ^678^VTGSDSEDE^686^ (SYDE1)
SYDE1	RhoB	RhoGTPase domain	Synaptic plasticity	Docking by Swarmdock^36^	–	RhoGAP
SYDE2	Pin1	WW domain	Regulation of NMDA receptor	ELM: LIG_WW3 Pin1 consensus motif^61,62^	5.67e−04	S/T-P motif ^60^SPPRS^64^
SYDE2	Smek1	Ser/Thr phosphatase4 regulatory subunit (EVH domain)	Neuronal differentiation	ELM: DOC_PP4 PP4 binding motif^60^	1.37e−03	FxxP motif, SLiM region ^158^FRDP^161^
SYDE2	Grb2	kinase	Cerebral cortical development	ELM: LIG_SH2_Grb2 Consensus pYxN motif by pull-down experiment^50^	3.18e−04	SH2 domain binding motif ^551^YINS^554^
SYDE2	Fbw7	SCF ubiquitin ligase complex, WD-40 motif	Neuronal differentiation	ELM: DEG_SCF_FBW7 Alignment of degron motifs^63^ ɸXɸɸɸTPPxS (ɸ: hydrophobic)	7.14e−04	TPxxS phospho-dependent degron ^552^INSPDNTPSLS^562^
SYDE2	Calcineurin	Ser/Thr phosphatase	Ca^2+^-dependent synaptic plasticity	ELM: DOC_PP2B_LxvP1^41^ πɸLxVP, SLiM region	2.30e−03	^179^SFLRPP^184^
SYDE2	Crk, Src	Kinase		ELM:LIG_SH3_3 Class I: RxxPxxP^56,57^	1.32e −02	^56^RQQVSPP^62^
SYDE1,2	Crk	Kinase		ELM:LIG_SH2_Crk/Nck, putative phosphorylation and conserved binding motif^59^	1.52e−03	SH2 domain binding motif ^112^YNPIP^116^ (SYDE1) ^372^YNPIP^376^ (SYDE2)
SYDE1,2	GSK3	Kinase	Regulation of synaptic clustering	ELM: MOD_GSK3_1, D_2_P_2_, conserved putative phosphorylation motif with DmRhoGAP100F^58^	2.68e−02	^228^GYLSDGDS^235^ (SYDE1) ^619^GYLSDGDS^626^(SYDE2)
SYDE1,2	RhoA	RhoGTPase domain	Regulation of dendritogenesis	Docking by Swarmdock^36^	–	RhoGAP

Protein	Interactor	Domain architecture	Signaling pathway	Experimental condition	Ref	Interacting region
SYDE1	Munc18	Sec1-like domain	Presynaptic differentiation	coIP	^3^	Disordered domain
SYDE1	Liprinα2	Coiled-coil, SAM domain	Presynaptic differentiation	coIP	^3^	Disordered domain
SYDE1	Ruk/CIN85	SH3 domain	Control of Rho-family GTPases	Pull-down, LC–MS/MS	^69^	–
SYDE1	XPO1	ImportinβN domain, Nuclear export (exportin1, XPO1 domain)	Nuclear export to the cytoplasm	High through-put AP-MS, putative nuclear export signals*	^80^	L^536^RLVSS^541^* L^713^KDFDALILDLERELS^728^*
SYDE1	ARHGAP28	RhoGAP domain	RhoGAP activity, Actin filament	coIP, High through-put AP-MS	^12^	–
SYDE2	PLEKHG3	DH domain, PH domain	RhoGEF activity	High through-put AP-MS	^12^	–
SYDE2	calcineurin	Serine/Threonine phosphatase	Activity-dependent dendritogenesis	Pull-down, ITC	^41^	SYDE2 232–289 ^235^RVLSVP^240^
DmSyd1	Mtl	Small GTPase Rho	Rho-GTPase activity	Pull-down	^4^	RhoGAP
DmSyd1	Rac1, Rac2	Small GTPase Rho	Rho-GTPase activity	Pull-down	^4^	RhoGAP
DmSyd1	RhoA, RhoL	Small GTPase Rho	Rho-GTPase activity	Pull-down	^4^	RhoGAP
DmSyd1	Cdc42	Small GTPase Rho	Rho-GTPase activity	Pull-down	^4^	RhoGAP
DmSyd1	Neurexin-1	lamininG domain, Syndecan/Neurexin domain	Neurexin-1 recruitment to active zones Postsynaptic GluR2 localization	Y2H, Co-IP, FLAP	^7,81^	PDZ
DmSyd1	Bruchpilot (ELKS-related protein)	ELKS/CAST domain, coiled-coil	Presynaptic active zone organization Postsynaptic GluR2 localization	Y2H, coIP-MS/MS	^82^	PDZ, C-terminal (1-320aa, 1301-1844aa)

For prediction of Syde interactors we searched for putative domains and linear motifs putatively mediating the interactions detected in commonly cell-based model or in neurodevelopmental processes. We assume that a predicted Syde linear motif may be potential interaction site for a novel interactor if the known binding protein is categorized to a class of protein or contains the motif which is already known to bind the corresponding sequences in the Syde proteins (Table 1).

## Results

**Figure 1:**
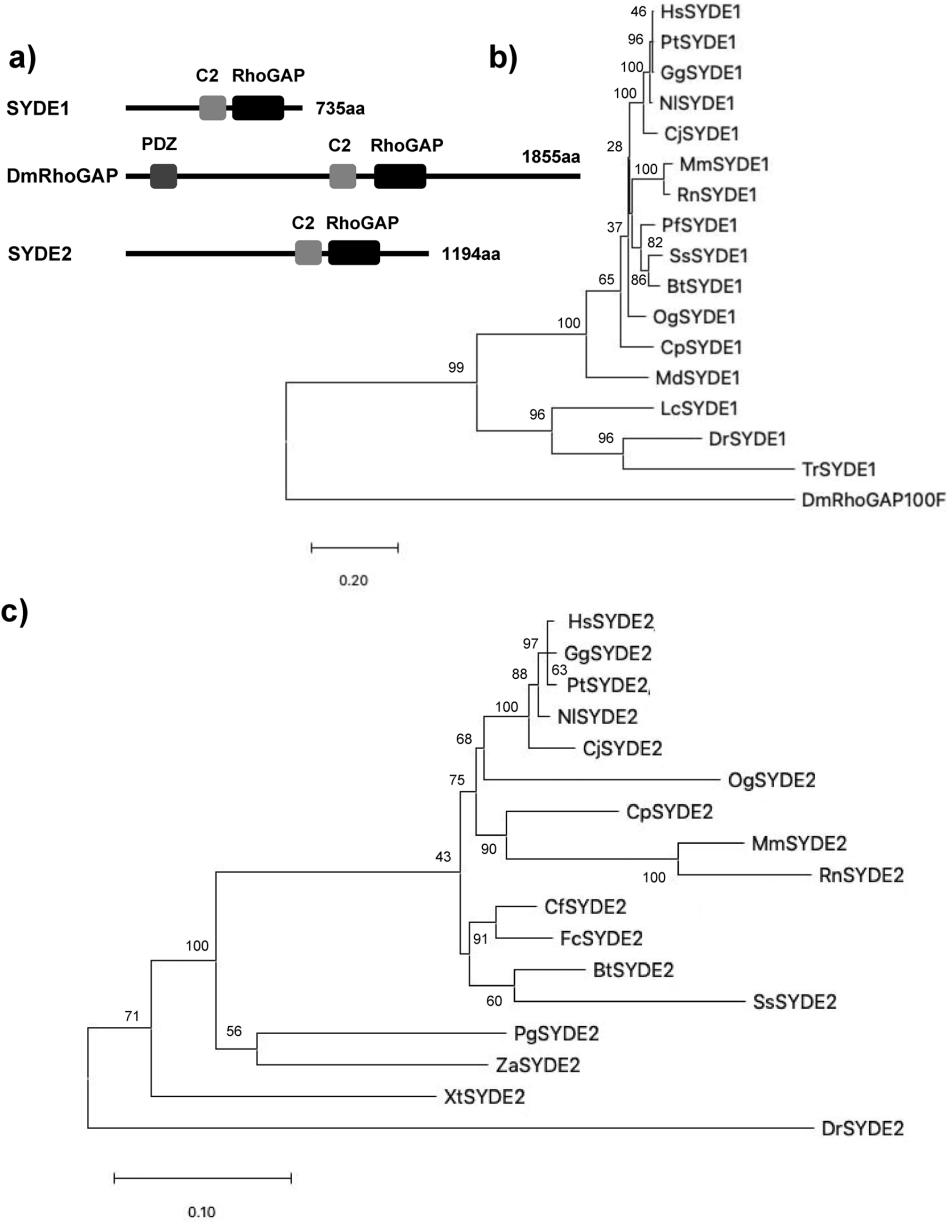
Phylogenic tree construction of SYDE1 and SYDE2. (**a**) Domain organization of Syde proteins. (**b**) The tree is constructed among vertebrate SYDE1 orthologs. Each cluster is organized as the following Syde1 genes: Bt: *B. taurus* (F1MXU4); Cj: *C. jacchus* (U3BZL8); Cp: *C. porcellus* (H0VZ67); Dr: *D. rerio* (E7FA87); Gg: *G. gorilla* (G3R4S4); Hs: *H. sapiens* (Q6ZW31); Lc: *L. chalumnae* (XP006002980); Md: *M. domestica* (F7F6S9); Mm: *M. musculus* (Q9DBZ9); Nl: *N. leucogenys* (XP030676439); Og: *O. garnettii* (H0XAB4); Pf: *M. putorius furo* (M3YXJ5); Pt: *P. troglodytes* (H2QFL6); Rn: *R. norvegicus* (D3ZZN9); Ss: *S. scrofa* (F1SAN7); Tr: *T. ruburipes* (XP02968941); DmRhoGAP100F: *D. melanogaster* RhoGAP isoformC. (**c**) For SYDE2 orthologs, each cluster is organized as the following Syde2 genes: Bt: *B. taurus* (NP001179527); Cf: *C. familiaris* (XP025273572); Cj: *C. jacchus* (F6R312); Cp: *C. porcellus* (H0UWH0); Dr: *D. rerio* (A0A0G2KK80); Fc: *F. catus* (M3XAW0); Gg: *G. gorilla* (G3QK10); Hs: *H. sapiens* (Q5VT97); Mm: *M. musculus* (E9PUP1); Nl: *N. leucogenys* (G1RG60); Og: *O. garnettii* (H0WYC5); Pg: *P. guttatus* (A0A6P9DB82); Pt: *P. troglodytes* (H2PZB3); Rn: *R. norvegicus* (NP001305229); Ss: *S. scrofa* (A0A4X1UF43); Xt: *X. tropicalis* (A0A6I8RMF6); Za: *Z. albicollis* (XP005482373).

**Figure 2:**
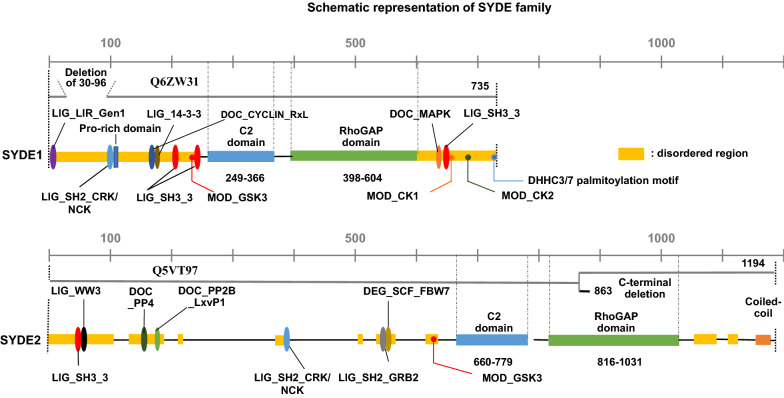
Domain architecture of SYDE proteins based on their sequence analysis. Both human SYDE proteins are characterized by disorder domain (yellow), C2 (light blue) and RhoGAP domain (green) that are indicated as sequence boundaries of human SYDEs (SYDE1: Q6ZW31; SYDE2: Q5VT97). Conserved linear motifs among vertebrates and mammals for SYDE1 and SYDE2, respectively, are represented as the following according to Dinkel et al*.* (2016): Canonical LIR motif required for ATG8-mediated autophagy (LIG_LIR_Gen1) (purple oval); Crk and Nck SH2 domain binding motif (LIG_SH2_CRK/NCK) (light blue oval); 14-3-3 interaction motif (LIG_14-3-3) (brown oval); Cyclin/CDK binding motif (DOC_Cyclin_RxL) (dark blue oval); PPR-specific WW domain (LIG_WW3) (black oval); a docking motif mediating interaction with Erk1/2 and p38 subfamilies of MAP kinases (DOC_MAPK) (orange oval); calcineurin docking motif (DOC_PP2B_LxvP1) (light green oval); FxxP-like docking motif recognized by PP4 holoenzyme (DOC_PP4) (dark green); Phsopho-dependent degron recognized by FBW7 Fbox proteins (DEG_SCF_FBW7) (dark yellow oval); Casein kinase (CK1) phosphorylation site (MOD_CK1) (orange dot); CK2 phosphorylation site (MOD_CK2) (dark green dot); DHHC3/7 palmitoylation site (green dot); GSK3 phosphorylation site (MOD_GSK3) (red dot); SH3 binding site (LIG_SH3_3) (red oval); Grb2-like SH2 domains binding motif (LIG_SH2_GRB2) (gray oval). Alternative SYDE isoforms are indicated and the SYDE1 isoform Q6ZW31-2 (668 residues) is missing the N-terminal region (30–96). SYDE2 isoform Q5VT97-2 (863 residues) is truncated at residue 849 with substitution in the region from position 850 to 863 (LSYYGSLLLPLLID).

**Figure 3:**
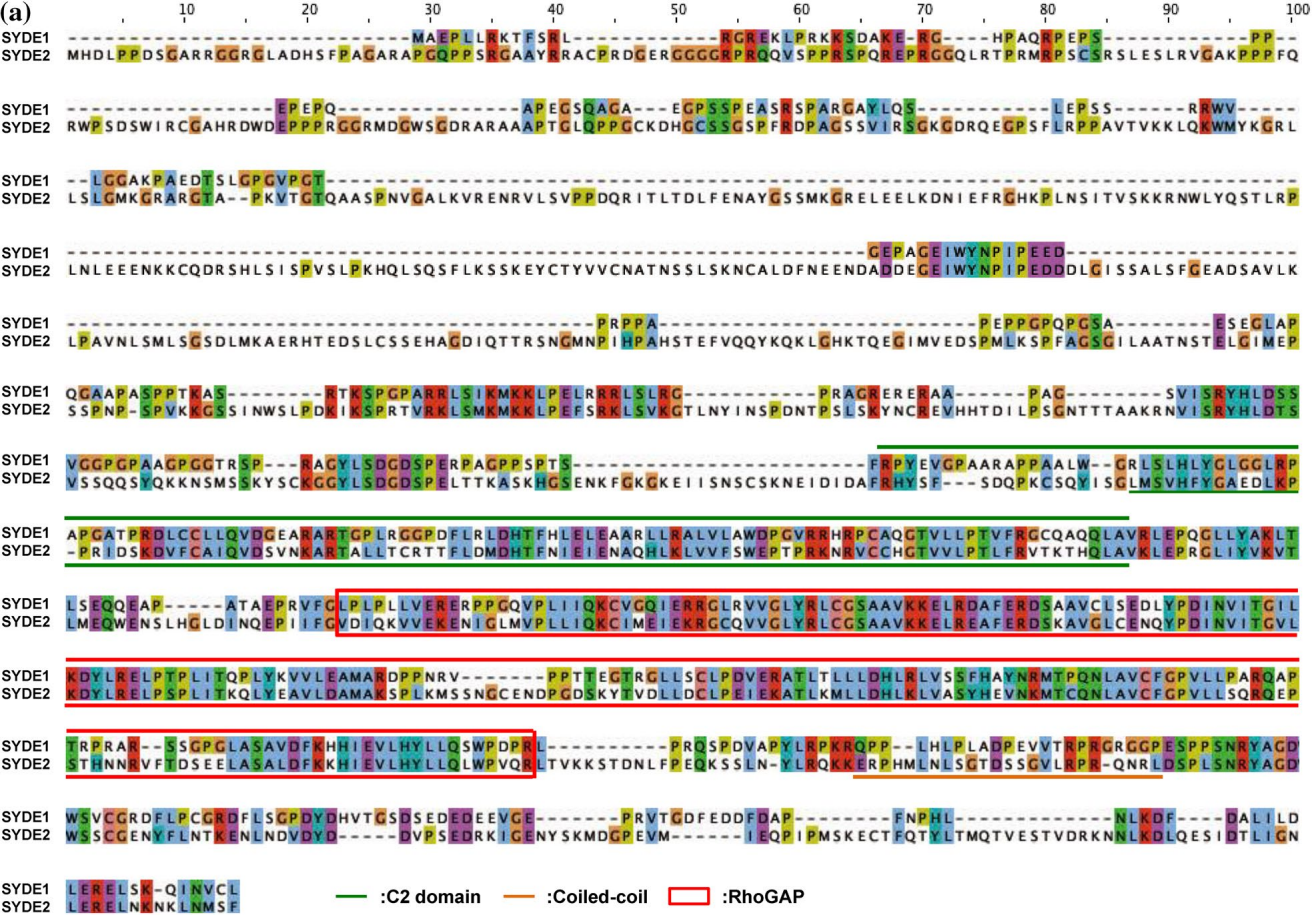

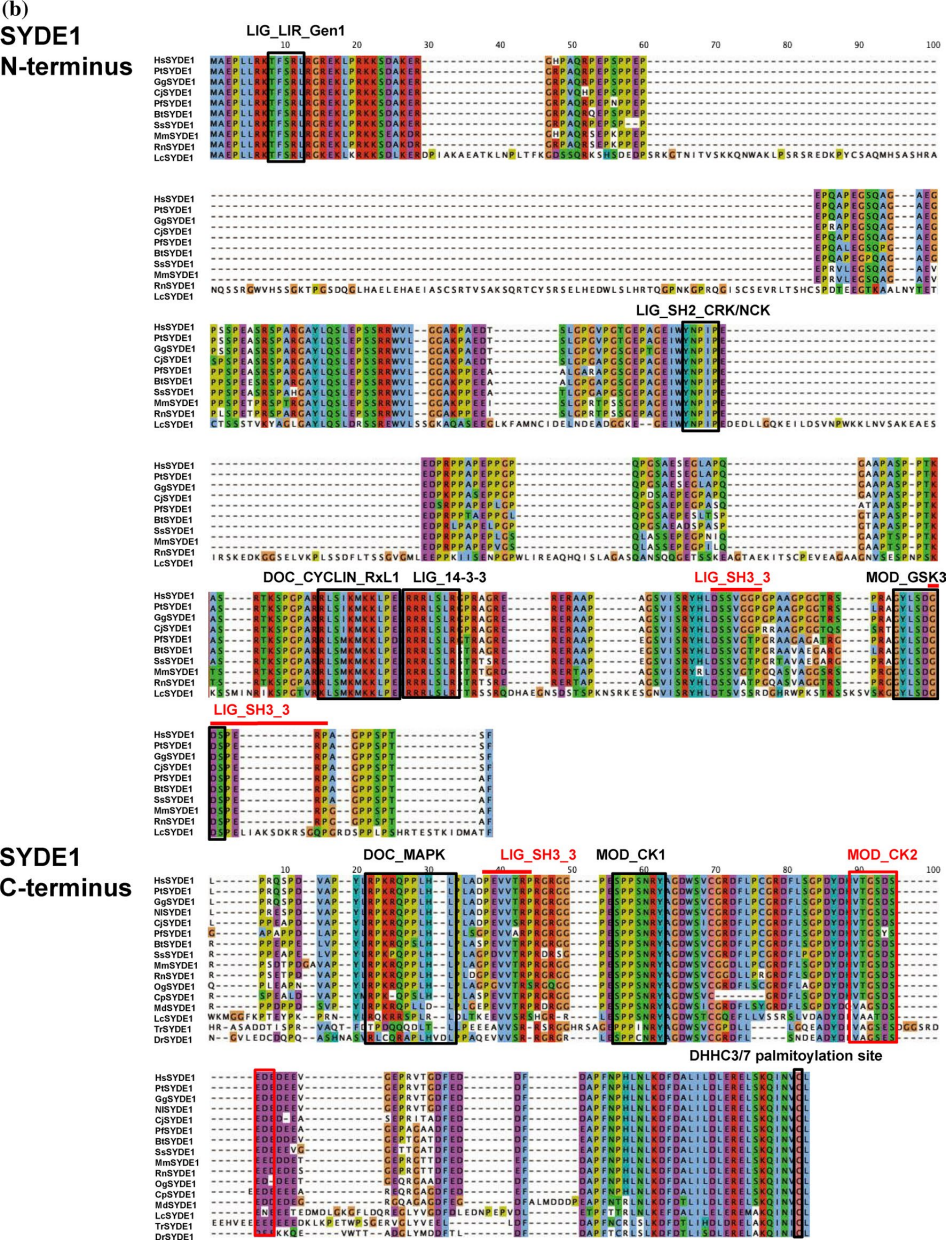

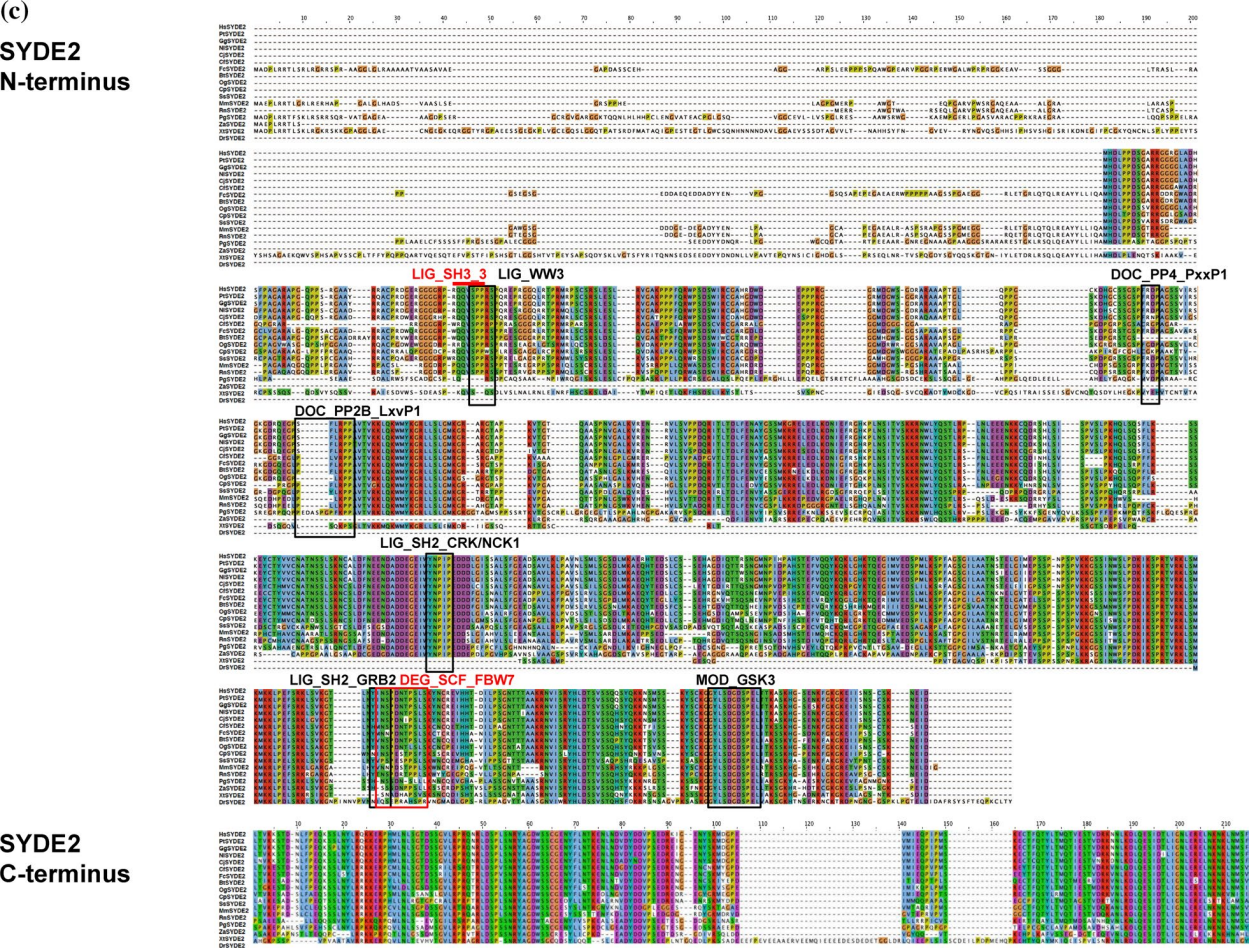
Multiple alignments of SYDE proteins. Color code is based on Clustalσ scheme and linear motifs are identified by ELM and D_2_P_2_ database Anchor definition^22^. (**a**) Alignment of SYDE1 and SYDE2. (**b**) Syde1 N-terminus and C-terminus. (**c**) Syde2 N-terminus and C-terminus. The signatures indicate: LIG_SH2_CRK/NCK: Crk and Nck SH2 domain binding motif; LIG_14-3-3; 14-3-3 binding motif; DOC_Cyclin_RxL: Cyclin/CDK binding motif; LIG_WW3: PPR-specific WW domain; DOC_MAPK: a docking motif for interaction with Erk1/2 and p38 subfamilies of MAP kinases; DOC_PP2B_LxvP1: calcineurin docking motif; DOC_PP4: a docking motif recognized by PP4 holoenzyme; MOD_CK1: CK1 phosphorylation site; MOD_CK2: CK2 phosphorylation site; MOD_GSK3: GSK3 phosphorylation recognition site; LIG_SH2_GRB2: Grb2-like SH2 binding motif; LIG_SH3_3: SH3 binding motif; DEG_SCF_FBW7: Phsopho-dependent degron recognized by FBW7 Fbox proteins; LIG_LIR_Gen1: LIR motif for ATG8-mediated autophagy.

Syde proteins have emerged as critical Rho-family regulators in neuronal and embryonic development. However, their physiological roles and molecular mechanisms in vertebrates are not well known. Therefore, a computational analysis of the SYDE-family proteins was performed by comparing the primary structures of *Drosophila* and vertebrate orthologs to explore their regulatory and catalytic functions. Homology search using InterproScan identified the C2 and RhoGAP domains that comprised 118 and 207 residues in SYDE1, and 120 and 216 residues in SYDE2, respectively. These predicted regions are highly conserved between vertebrate Syde1 and Syde2, although each N- or C-terminal sequences is quite diverse (Figs. 1, 2, 3). In addition to this unique evolutionary variance observed in the disordered Syde domains, crystal structure is not available for any Syde-family protein (Fig. 3a). Disordered regions are a particular stretch of amino acid patterns or conserved regions with predicted linear motifs that mediate modification by phosphorylation or provide an interface to specific binding partners for signaling (Fig. 2). Syde1- and 2-binding proteins were examined by BioGrid and IntAct and listed by curating additional relevant literature (Table 1, lower table). These findings were used to expand for assembling the Syde interaction network (Table 1). The SYDE interactors were selected as the following procedures. Putative interaction motif scoring e-value lower than 1.0 × 10^–2^ was selected based on its location within the disordered domain mapped by ELM, D_2_P_2_ and Anchor, and phosphorylation site was selected based on the disordered propensity and predicted site assigned by Phosphosite. Then, the motif conservation through mammalian SYDE1/2 orthologs was also taken account to select the functional interaction sites. As for evolutional aspects, all the listed motifs were conserved and could be functional in MdSYDE1 (M. domestica), whilst casein kinase II phosphorylation site (MOD_CK2) and Grb2 binding motif (LIG_SH2_Grb2) lacked in LcSYDE1 and FcSYDE2, respectively as indicated in ELM search (Fig. 3b,c). In contrast, ZaSYDE2 was devoid of most of the interaction and phosphorylation motifs (Figs. 1c and 3c). The putative functional units are described by predicted domains or elements separately as follows.

### N-terminal regulatory regions

The intrinsically disordered region resides in the N-terminus, especially with a more extended form in Syde1 than in Syde2. This portion of mammalian Syde2 proteins shows a relatively low identity between its paralogs, suggesting functionally divergent roles (Fig. 1). The lower organisms contain a PDZ domain in the middle region with the N-terminal side of C2 domain. However, the corresponding motif does not reside in the vertebrate Syde proteins (Fig. 1). An alignment of the N-terminus of the vertebrate Syde1 highlights the highly conserved regulatory motifs including the SH2 binding region. But vertebrate Syde2 contains evolutionarily conserved motifs with variable regional sequences outside of the motif. The ATG8 family interacting LIR motif and Crk docking motifs are located at the N-terminus of Syde1. The 14-3-3zeta and cyclin B/Cdk1-binding site are located within the C-terminal side of the disordered domain (Fig. 3b), and the GSK3 phosphorylation site resides within the linear motif. The Syde1 isoform2 (Q6ZW31-2) lacks 67 residues in the disordered domain but preserves all of the predicted motifs. In contrast, the N-terminal portion of Syde2 has calcineurin docking LxVP motifs and the motif in the marginal region of the disordered domain was experimentally proven to interact with calcineurin^41^ (Figs. 2, 3c). Pin1 specifically binds the WW recognition motif represented by phosphorylated serine or threonine residues preceding a proline, and SYDE2 proteins contain a group III/IV motif and SH3 binding region at the N-terminus. The WW domain for Pin1 recognition and Smek1 binding site for serine/threonine protein phosphatase 4 (PP4)-mediated regulation are conserved among mammalian Syde2 proteins but not in birds, reptiles, amphibians and fish (Fig. 1, *Z. albicollis*, *P. guttatus*, *X. tropicalis*, and *D. rerio*). Clusters of short-length disordered domains are located in the middle of Syde2 (Figs. 2, 3). Crk and Grb2 binding motifs reside separately in the disordered regions where highly conserved sequences among vertebrates and C-terminal variable regions are sequentially organized.

### The RhoGAP domains of SYDE1/2 and *Drosophila* Syd1

**Figure 4:**
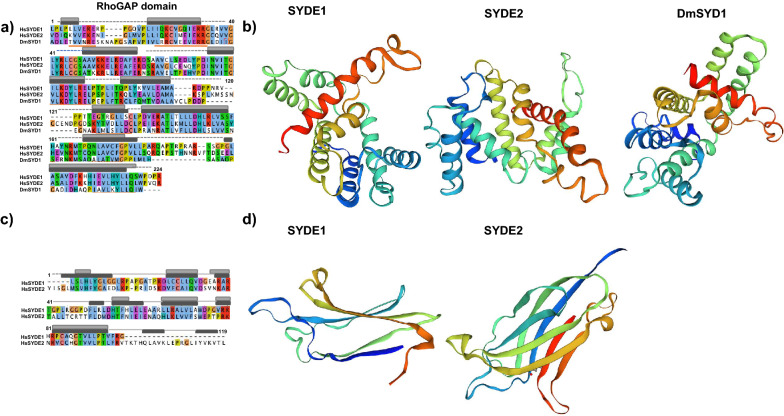
Overview of SYDE RhoGAP domains and structural RhoGAP models. (**a**) Consensus sequences of SYDE1 RhoGAP domains are shown in the panels. Secondary structures of human SYDE1 are indicated above the sequences (α-helix: gray column for SYDE1 and dark gray column for SYDE2; coiled-coil: gray dotted line) and α-helix of DmSyd1 is represented by horizonal orange line below the sequences. (**b**) Cartoons of SYDE RhoGAP domains are colored from the N-terminus (blue) to C-terminus (red). (**c**) Consensus sequences of SYDE C2 domains and secondary structures of human SYDE1/2 used for structure prediction are indicated above the sequences (β-sheet: gray column for SYDE1 and dark gray column for SYDE2). (**d**) Structure of SYDE C2 domains are colored from the N-terminus (blue) to C-terminus (red) by UCSF Chimera. The three-dimensional structures were visualized using UCSF Chimera software version 1.15 (http://www.cgl.ucsf.edu/chimera).

The RhoGAP domain of human SYDE1 and SYDE2 proteins show 57.1% sequence homology with each other. These two RhoGAP domains have similar levels of lower sequence homology to the corresponding domain of *Drosophila* Syd1 (Q9V7SV) (SYDE1: 46.3%; SYDE2: 45.0%) (Fig. 4a). The RhoGAP domains of both Syde1 and Syde2 were modeled separately based on tertiary template selection. The HHPred search and potential template candidates by trRosetta and I-Tasser selected human MgcRacGAP protein (PDB code: 5c2k) for both Syde RhoGAP domains, and ArhGAP2 (N-chimerin PDB code: 3cxl) was selected as the best model for *Drosophila* Syd1 (Fig. 4b, Supplementary Fig. S1, Supplementary Table S2). Evaluation of predicted template-based model was performed by Procheck and ERRAT^35^ (Table 2). Despite the insertion between Syde and template sequences, the secondary structural elements of the RhoGAP domains in both Syde proteins were superimposed well to MgcRacGAP, which is organized chiefly with helix and turn structures. MgcRacGAP functions as a GAP activity toward Cdc42 and RhoA and the structures of the docking complexes with MgcRacGAP were experimentally resolved^42^. The structures of the catalytic core were well structurally superimposed. Both RhoGAP-Rho interface showed well-matched structures by forming a conserved arginine finger in the GAP domain of Syde proteins bridging the β and γ phosphate groups of GTP required for its hydrolysis and the cysteine residues located at the substrate interfaces of both proteins are substituted by Ser387 in MgcRacGAP^43^ (Fig. 4b). Phosphorylation of serine 387 in MgcRacGAP is known to determine the substrate specificity towards RhoA rather than Rac1 and the replacement of this residue by aspartic acid reduces the RacGAP activity^44^. The 3D model quality evaluation of Syde1 and Syde2 revealed closely homologous structures, with QMEAN scores of 0.66 and 0.64, respectively, and *Drosophila* Syd1 is remotely homologous to the ArhGAP2 RhoGAP domain with QMEAN score of 0.74. Lower quality regions are located in both Syde1 and Syde2 as insertion between the sixth and seventh α-helices due to partially disordered loops; otherwise highly positional conservation is shown in the Syde proteins with MgcRacGAP (Supplementary Fig. S1). Both proteins showed similar α-helical distribution patterns, which could mediate the similar recognition patterns of Rho-family substrates. The SYDE2 isoform (Q5VT97) lacks residues 864–1194, missing RhoGAP and the coiled-coil domains (Fig. 2).

**Table 2:** Evaluation of predicted structural models. The model based on template-based prediction was calculated. Ramachandran plots showed several disallowed residues in RhoGAP and HsSYDE2 C2 domain except HsSYDE1 C2 domain with completely favored score in all the predicted region. Each predicted model showed high score in overall quality factor for non-bonded atomic interactions by ERRAT.

	HsSYDE1 RhoGAP (5c2k)	HsSYDE1 C2 (1djx)	HsSYDE2 RhoGAP (5c2k)	HsSYDE2 C2 (5iz5)	DmSyd1 RhoGAP (3cxl)
Ramachandran Plots (favored)	98.2%	100%	97.8%	98.2%	98.3%
Ramachandran Plots (disallowed)	0.6%	0.0%	0.5%	0.9%	0.6%
ERRAT (overall quality factor)	94.09	77.10	98.96	82.69	92.67

Highly conserved residues among RhoGAP family proteins (Arg436_,_ Lys476, Arg480, Met548, Asn552, and Pro559 in Syde1 numbering) are conserved with MgcRacGAP^15,42^. Among the vertebrate Syde1 and Syde2 orthologs, these residues in the Rho-family proteins are highly conserved, although some exceptional cases exist (Syde1 in *D. rerio* and Syde2 in *O. garnettii*, E7FA87 and H0WYC5, respectively). Structural analysis with sequence comparison revealed that both Syde1 and Syde2 with MgcRacGAP displayed completely matched orientation of the α-helices that contained the putative conserved residues interacting with Rho family protein (Fig. 4b, Supplementary Fig. S1). Cdc42-bound MgcRacGAP has different regional structures around the C-terminal α-helices 2 and 3 sequentially flanking the catalytic domain compared to its RhoA-bound model (PDB code: 5c2k and 5c2j). The conserved residues around the arginine finger and α-helix 9 in ArhGAP2 for Rho-family protein binding are Gly301/Arg304 and Met413/Asn417/Val421/Pro424, respectively, and these residues are conserved with the corresponding residues of DmSyd1 RhoGAP domain^15^. In the case of DmSyd1, lower quality of matched region exists in the loop region between α-helix1-2 and 6–7, which is remotely located at the putative interface of the Rho-family protein (Supplementary Fig. S1). *Drosophila* Syd1 variable residues in the α-helix 9–10 organizing interface for Rho-family proteins do not match with the corresponding residues in ArhGAP2, but similar catalytic core EIE sequence within the α-helix2 of ArhGAP2 resides in DmSyd1 as EVE, which is highly conserved among chimerins^45^. Thus, the substrate interface of DmSyd1 may be conserved with ArhGAP2 in addition to structural matching.

**Figure 5:**
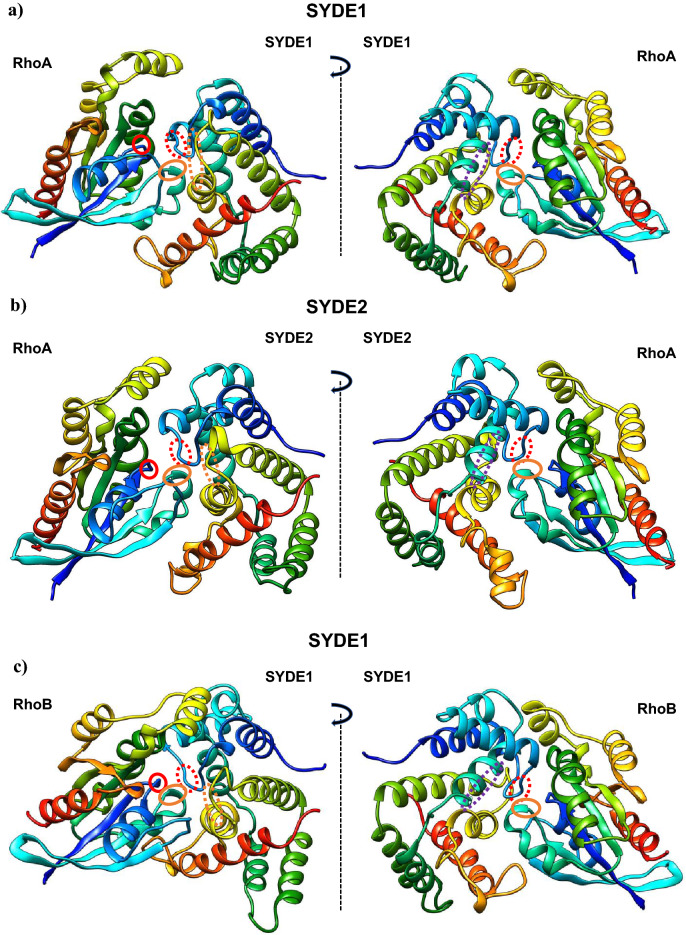

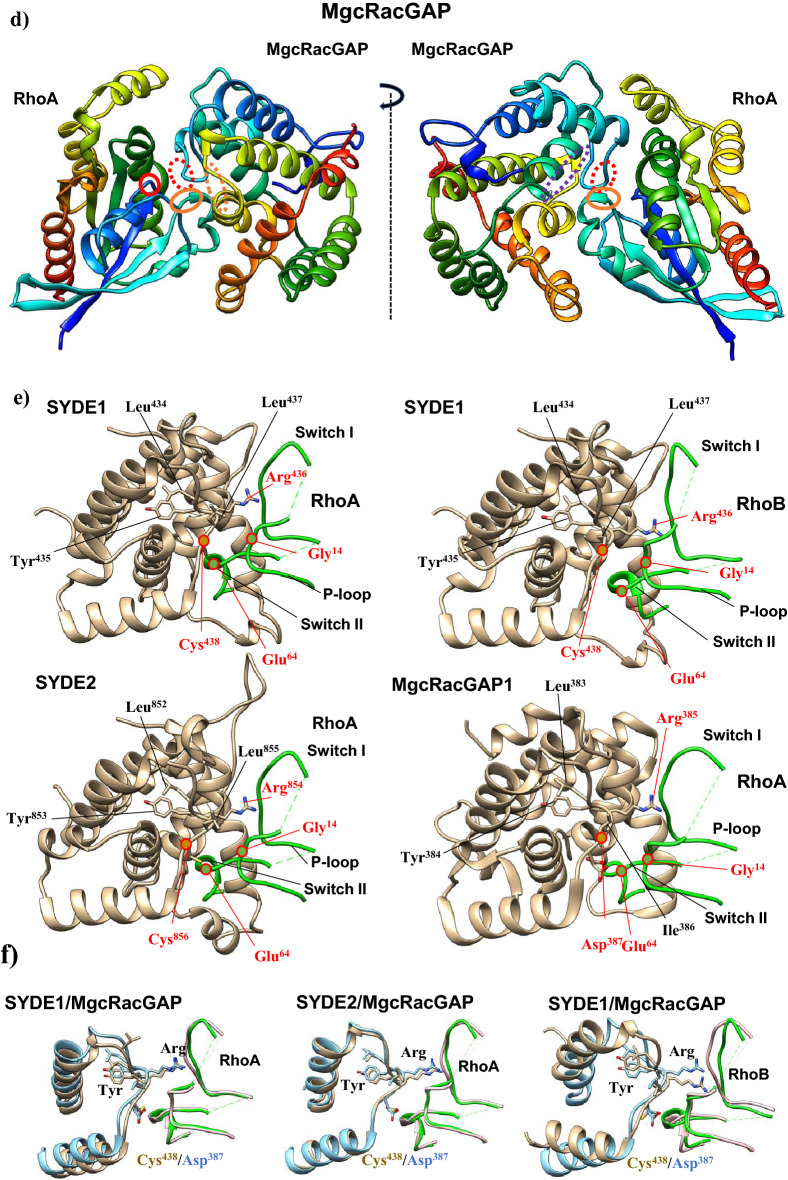

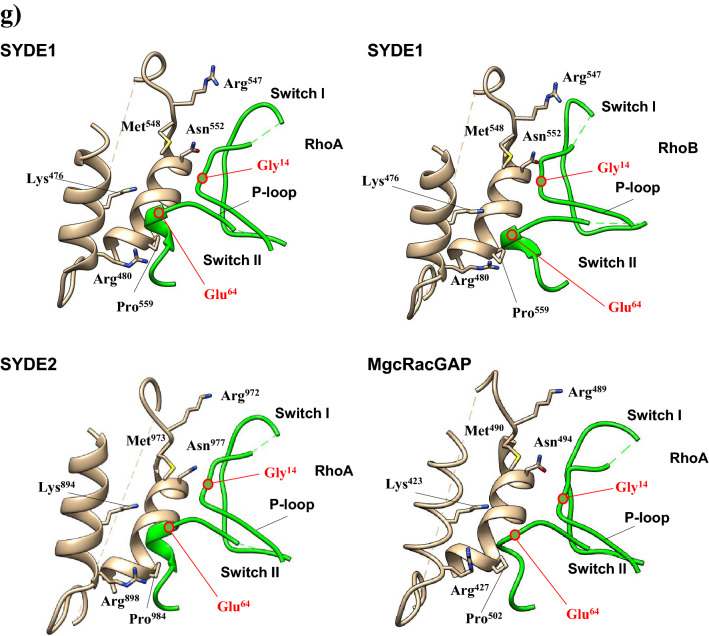
Model of SYDE1 and SYDE2 RhoGAP domain complexed with RhoA. RhoGAP domains of SYDE1 (**a**) and SYDE2 (**b**) bound to RhoA, and SYDE1 RhoGAP (**c**) bound to RhoB were modeled by SwarmDock program. Structure of MgcRacGAP/RhoA complex is shown (**d**). Conserved regions consist of arginine finger (red dotted circle), positively charged interface (purple dotted oval) and hydrophilic interface (orange dotted oval) in RhoGAP domains face to switch II (orange oval) and P-loop (red circle) of RhoA are indicated. Predicted structure of the substrate recognition sites specifically interacting with P-loop and Switch I and II are indicated (**e**–**g**). The side-chains of the conserved residues among the RhoGAPs are shown on the cartoon (**e**–**g**). (**e**) Arginine finger locates between second and third α-helices and cysteine and arginine tether to the interface formed by glycine and glutamic acid of RhoA or RhoB (red-edged circles). (**f**) Each modeled arginine finger in the RhoGAP complex with RhoA or RhoB is superimposed to the corresponding region of MgcRacGAP structure (PDB: 5c2k). RhoGAP domains of SYDE1/2 and MgcRacGAP are shown as the gold and light blue, respectively. The bound-RhoGTPases to SYDE1/2 and MgcRacGAP are colored as green and pink, respectively. (**g**) RhoGAP domain and P-loop and SwitchI/II region of RhoGTPases are colored as gold and green, respectively. Side chains of the conserved positively charged residues and conserved residues organizing hydrophilic regions show similar orientation on the ternary helical structure to the corresponding residues of the template by forming the Rho binding interfaces. Cartoon of each model was manually retrieved and indicated as colored molecules separately from the predicted docking structure by Chimera software.

Molecular docking of Syde1 and Syde2 was performed with RhoA and cdc42 using the SwarmDock program to examine the fitting of the predicted Syde-family proteins to the substrate recognition^36^. Both predicted Syde1 and Syde2 structures were fitted well to RhoA recognition with a RMSD of 0.760 and 0.657 Å, respectively, reflecting a highly conserved structural interface with the RhoGAP template (Fig. 5a,b,d). The catalytic arginine finger (Arg436 and Arg854 in Syde1 and 2) was sandwiched by glycine in P-loop and glutamate in the switch II region of RhoA (Fig. 5e,f). Moreover, a conserved positively charged interface (Lys476 and Lys894; Arg480 and Arg898 in Syde1 and 2) and hydrophilic regions (Met548 and Met973; Asn552 and Asn977 in Syde1 and Syde2) in the fifth and ninth α-helices, respectively, were close to the RhoA interface (Fig. 5g). RhoB was also selected as SYDE1 binding protein by SwarmDock program with analogy to the predicted SYDE1-RhoA interaction model (Fig. 5c,e,f). No docking model was selected through the molecular docking search for the interaction of cdc42, RhoC, RhoD, and RhoG with the Syde proteins.

### The C2 domain of the SYDE1/2 proteins

The InterPro search and comparative sequence analysis indicated that the N- and C-terminal boundary of the regions of Syde1 C2 domain were less conserved among orthologs than the corresponding regions of Syde2 (Fig. 4c). The human Syde1 and Syde2 proteins show 42.6% sequence homology in the C2 domain. These C2 domains were modeled separately based on tertiary structure through HHPred search. The best selection was obtained with PLCδ1 protein (PDB code: 1dji) for Syde1 and GIVD cytosolic phospholipase A2 (cPLA_2_δ, PDB code: 5iz5) for Syde2 (Fig. 4d, Supplementary Fig. S2). The 3D model quality of Syde1 and Syde2 C2 domains was evaluated, and remotely homologous structures with QMEAN scores of 0.55 and 0.48, were obtained, respectively.

The PLCδ1 C2 domain binds Ca^2+^ in a phosphatidylserine (PS)-dependent manner. The binding involves Ca^2+^ binding loops (CBR1-3) located on the same end of the β-sandwich structure with topologically P-family type II^46^. The CBR3-like region in Syde1 between Asp337 and Arg344 corresponded to Asp708 and Asp714 in PLCδ1 and was oriented toward the outside; other regions including the CBR1 and CBR2 were superimposed well to the PLCδ1 C2 domain. Asp288 and Asp337 in Syde1 matched the corresponding residues Asp653 and Asp708 as opposed to two Ca^2+^. However, the other residues in Syde1 were not conserved with those located in the loop region mediating Ca^2+^ binding and PS binding in PLCδ1^47^. Both GIVB and GIVD cPLA_2_ C2 domains have a high affinity for 1-palmitoyl-2-arachidonyl-sn-glycerol-3-phophocholine (PAPC) in the presence of Ca^2+^. The basic residues (Lys24/Arg49/Lys52, Lys78 and His44/His82) in the GIVB cPLA_2_ C2 domain form the interface for binding to PAPC. Although the residues in GIVB are not conserved with GIVD cPLA_2_, GIVD possessed a higher affinity for PAPC than GIVB cPLA_2_ when tested in the in vitro vesicle binding assay^48^. Moreover, the structural interface organized with these residues of Syde2 C2 region was superimposed well to GIVD cPLA_2_ except for the loop region containing Lys24 of GIVD cPLA_2_^48^.

### C-terminal regulatory region in Syde1

Syde1 has a C-terminal disordered domain conserved among mammalian orthologs. The Cys734 palmitoylation site resides at the C-terminus in vertebrate SYDE1 but not in *Drosophila* and worm Syd1^26^ (Figs. 2, 3b). Reversible palmitoylation by Golgi-localized DHHC3 allows Syde1 to shuttle between intracellular compartments and plasma membranes. The C-terminal Syde domain contains phosphorylation sites by casein kinases and the p38 subfamily MAPK and calcineurin interaction sites which are highly conserved among mammalian orthologs. Putative SH3 binding motif is located in the middle of the highly conserved disordered domain.

### Syde1 and Syde2 networks

**Figure 6:**
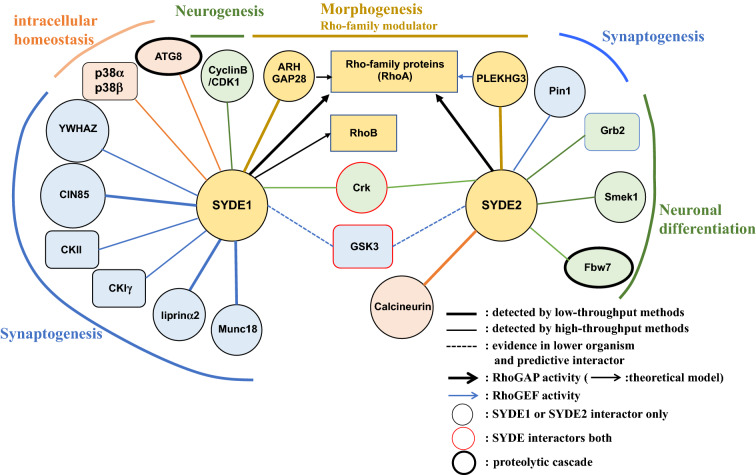
SYDE protein interaction network. SYDE binding partners identified by low-throughput data (bold line), high-throughput protein–protein interaction data or linear motifs prediction (thin line), and the interactions mediated by conserved sequences as linear motif that are predicted to be associated with binding partner proven in lower organism (dotted line). Interactors are represented by different shapes based on their molecular function: protein kinases (rectangles); degradation machineries (bold-lined ovals); adaptor proteins or phosphatases (circles). Interactor is colored and categorized by neuronal regulatory function as follows: Rho-family modulators (yellow); synaptogenesis (light blue); intracellular homeostasis (light pink); neuronal differentiation (green). SYDE interactor both is marked by red edges and either SYDE1 or SYDE2 only binding partner is represented by black edges. *CKIγ* casein kinase Iγ, *CKII* casein kinase 2.

Eleven Syde1 and eight Syde2 interactors were manually curated, and four SYDE1 and two SYDE2 binding proteins were directly retrieved from literatures (Fig. 6, Table1). Most of interactors belonged to a category of proteins that recognized a conserved linear motif located in the disordered regions. XPO1 is indicated as an Syde1 complex by high-throughput evidence, and two regions are predicted as the potential nuclear export signatures by XPO1 in Syde1 (Table1). S-palmitoylation of Casein kinase 1γ mediates phosphorylation and intracellular transport of Lyn at Golgi apparatus^49^. Golgi-localized DHHC3 specifically palmitoylates Syde1 and casein kinase 1γ might phosphorylate palmitoylated SYDE1^26^. ELM search selected the SH2 binding motif YINS in SYDE2 that was matched with typical Grb2 binding motif validated by pull-down experiment^50,51^. MAPK binding D-motif resides at the C-terminal region in SYDE1, and the sequence has similarity with MKK-type phosphorylation motifs catalyzed by p38^52^. ELM search hit two 14-3-3 binding motifs in SYDE1, and several interactors such as Pctaire1 kinase possess similar RLSLP sequence mediating the interaction^53^. 14-3-3ζs are dimeric with diagonal symmetry wherein two phosphate-binding sites lie in diagonally opposite position when they form a complex with kinases such as the CaMK and AGC family, including PKA. Cin85 is enriched in the brain and localized in the postsynapse, and associated with endocytosis regulators such as endophilins and potential SH3 interaction sites were indicated in SYDE1 (Fig. 3b). SYDE1 possesses RxL docking motif for cyclin binding (DOC_Cyclin) at the N-terminal disordered domain. The sequence was matched to consensus Cdk1/CyclinB complex binding motif identified by Arg/Lys-scanning oriented peptide libraries^54^. The LIR (LC3-interacting region) motif consists of core W/F/I-X-X-ɸ (ɸ: L, I, F) sequence required for selective autophagy. Structural studies indicate the following key features: the aromatic and hydrophobic residues interact with distinct hydrophobic pockets of ubiquitin-like modifiers and an acidic or a phosphorylated Ser/Thr immediately upstream of the core sequence promotes the interaction^55^. The LIR motif in SYDE1 satisfied the definition that was categorized as F-LIR based on the aromatic residue. Noncanonical class I SH3 binding motif RQQVSPP resides at the N-terminus of SYDE2 and brain-specific ArhGAP32 contains the similar motifs that mediate interaction with Crk^56,57^. GSK3 antagonizes Syd1 and liprin ortholog Syd2 pathway in synaptic vesicle clustering in *Drosophila* and sequence alignment suggested that the potential conserved phosphorylation sites lie within the linear motif in both the SYDE1 and SYDE2 as described^58^ (Table 1). Conserved Crk binding YNPIP sites reside in both SYDE1 and SYDE2, and this Tyr phosphorylated motif shows similarity in the patterns with Crk binding sites in Dab1 and other substrates^59^. Calcineurin binding πɸLxVP motif containing polar and polar/hydrophobic residues at position − 2 (π) and − 1 (ɸ), respectively, was selected based on ELM search and its location in SLiM domain^41^. SYDE2 contained SFLRPP and RVLSVP sequence matched the πɸLxVP motif by ELM search although the motif selected by ELM in SYDE1 did not fulfill the definition. ELM search hit Smek4 as the SYDE2 interactor and centrobin fragment containing FRVP showed the similar motif patterns and high affinity to PP4 by ITC and coIP experiments^60^ (http://slim.icr.ac.uk/motifs/pp4/). As for Pin1 binding motif, multiple S/T-P motifs mediate the interaction in the case of gephyrin and PSD95, and one S/T-P motif sufficiently functions as Pin1-mediated neuroligin2 binding to gephyrin^61,62^. SYDE2 contains the typical S/T-P motif similar to the Pin1 binding region in the synaptic components at the N-terminus and other putative Pin interaction sites (DOC_WW_Pin1_4) were omitted due to low probability in ELM score. Fbw7 binding consensus degron is represented by ɸXɸɸɸTPPXS (ɸ: hydrophobic residue, X: any amino acid). Phosphorylated Thr and Ser residues interact with WD domain of Fbw7 and these residues were conserved in Fbw7 binding motif in SYDE2^63^. ArhGAP28 has actin-associated RhoAGAP activity with no Rac1 and Cdc42 selectivity; PLEKHG3 functions as RhoGEF in the brain. Both Syde1 and Syde2 have lower GAP activity toward Cdc42 than RhoA and Rac1, which was consistent with our prediction model^12^ (Fig. 5). Most Syde1 interactors such as liprinα2 and Munc18 are categorized as regulators of synaptogenesis including active zone homeostasis. In comparison, Syde2 binding proteins are primarily involved in neuronal differentiation signaling during embryonic brain development.

## Discussion

Rho GTPases are important for cell adhesion, motility, cytokinesis and contractile responses due to growth factor-induced reorganization of the actin cytoskeleton. Syde1 regulates synaptic exocytosis and its RhoGAP activity is a prerequisite for dendritogenesis. Although the in vivo function of mammalian Syde2 remains unknown, Syde2 isoform2 lacks most of the RhoGAP domain at the C-terminus, suggesting complex regulation of the Syde proteins. The homozygous nonsense C.1544C > G mutation (p.(Ser515*) homo) in Syde2 found in an intellectual disability patient generated a C-terminal truncated transcript devoid of the entire C2 and RhoGAP domain, suggesting a specific role for Syde2 in brain development^11^. It was reported that the C2 domain of Syde1 in addition to the N-terminal disordered domain has an autoinhibitory role for RhoGAP activity^3^; Therefore, the current study may provide the vertebrate-specific Rho-family substrate recognition by the Syde1 RhoGAP domain. Prediction and the structural quality of RhoGAP domains listed in Supplementary Table S2 satisfied the criteria as the potential template listed by trRosetta and I-Tasser. As for the C2 domain of SYDE1 and SYDE2, the potential templates were also selected in trRosetta predicting the structure by multiple sequential alignments as well as calculation of inter-residue orientation, but not in I-Tasser. The predicted model of RhoGAP in Syde1 and Syde2 applied to MgcRacGAP enabled the comparison of the structural interface for its RhoA and Cdc42 (PDB:5c2j), which have different regional conformations that may constrain substrate recognition. A flexible molecular docking search by SwarmDock selected the interaction of both predicted Syde1 and Syde2 with RhoA but not cdc42. The result was consistent with the substantial and extremely low GAP activity toward cdc42 in the RhoGAP assay system using FRET sensors^3,12^. Interestingly, RhoB was also selected as the SYDE1 substrate although the C-terminal regions of RhoA and RhoB show difference in primary sequences to each other. Neuronal regulation of RhoB activity by RhoGAP remains unknown and RhoB is known to regulate dendritic morphology and synaptic plasticity^64^.

The functional Syde protein network was expanded and categorized by integrating structure- and interactor-based prediction with high-throughput evidences: this included inferring the binding partners conserved in lower organisms. Most short linear motifs have a high chance of random occurrence and low specificity. However, stringent criteria were used for selection of interaction sites^65^. A predicted binding site and post-translationally modified residues must be conserved among orthologs and located in a disordered region. Therefore, motif patterns and phosphorylation sites were inferred through several methods^20–22,25^. As for the mammalian Syde proteins, the RhoGAP activity may be highly regulated by phosphorylation/de-phosphorylation and membrane targeting. Both Syde proteins may regulate synaptic plasticity and dendritic morphology with cooperative Ca^2+^-dependent synaptic regulation by Munc18-1 and liprin-α2, Palmitoylation regulates Syde1-specific membrane targeting and casein kinase Iγ might cooperatively regulate RhoGTPase’s activity at plasma membrane or presynaptic compartment^26,27,49^. Liprin-α2 and Munc18-1 are known to interact with the Syde1 N-terminal disorder domain and organize active zone complexes in presynaptic regions^3^ presumably in a competitive manner with RPTP-liprinα interaction^9^. Optimal 14-3-3 binding sites satisfy basic residues in positions − 3 to − 5 relative to the phosphorylated site and kinases in the AGC family, such as PKA/PKG/PKC and CaMK subfamilies, are most commonly implicated in the phosphorylation of the binding sites^53,66^. Therefore, Ca^2+^-dependent scaffold complex formation might be involved in the Syde1 interaction network. Crk and CIN85 containing SH2-SH3 and three SH3 domains, respectively, are known to regulate pre- and post-synaptic architecture colocalized with PSD95, synaptophysin, and Dock-180^67^. Syde1 and MgcRacGAP were identified by CIN85 interactors through the SH3-C region recognizing the PX(P/A)XXR motif, and Syde1 possesses potential SH3 binding sites as the consensus sequence matched with a variety of sequence patterns^68,69^. The predicted Syde1 interactor cyclin B/Cdk1 is involved in embryonic neurogenesis with the fate competency in opposition to cyclin D function^70^. Cyclins have cross-selectivity and Cyclin D recognizes RxL motif, therefore, SYDE1 might also interact with cyclinD. Casein kinase 2 localizes to the cytosol or membrane in neurons and phosphorylates PACSIN1, thus shutting off Rac1 hydrolysis in the process of dendritic spine formation^71^.

Neuronal Pin1’s prolyl cis/trans isomerase activity mediates NMDA dissociation from the PSD95 complex and structural arrangement of voltage-gated K^+^ channels upon neuronal excitability^72^. Syde2’s RhoGAP activity might be regulated by Syde2-specific Pin1 binding through the N-terminal disorder region in contrast to the autoinhibitory conformational changes in SYDE1. The Smek1 and PP4 catalytic subunit PP4c are required for neurogenesis^73^ and Grb2 is recruited to Ca^2+^-dependent kinase Pyk2 and ErbB2/3 receptors upon Nrg1 signaling during cerebral cortical development^74^. PLEKHG3 is highly expressed in the brain and microdeletion, including the gene, which is known to cause mild mental retardation with spherocytosis^13,75^.

Crk is known to be recruited to Dab1 upon Reelin signaling^76^ and may recognize Syde1 and Syde2 proteins through the SH2 domain. Liprin2 converging with PP2A phosphatase are regulated by GSK3β phosphorylation as downstream signaling cascades of Syd1 during neurogenesis in *Drosophila*^58^. Predicted phosphorylation by GSK3β may be required for both mammalian Sydes signaling involved in the exocytosis of synaptic vesicles (Table 1)^58^. The LxVP binding pocket interacts with calcineurin for comprehensive analysis of its substrates^41^. The current search additionally predicted the N-terminal calcineurin binding motif in the Syde2 disordered domain. Linkage of calcineurin with Syde2 implies synaptic activity-dependent AMPA and NMDA receptor function^77^.

Lastly, Syde-family proteins may be regulated by autophagy and proteasomal degradation during neuronal development. LC3/GABARAP protein noncovalently binds to the canonical ATG8 family-interacting motif (AIM), a core motif consisting of W/F/I-X-X-L/I/V, where any two amino acids flank the aromatic and hydrophobic amino acid during the process of phagophore formation in the autophagy pathway^63^. Syde1 might be regulated by ATG8-family-mediated signaling through the AIM motif by coupling with a reversible DHHC3/7-mediated palmitoylation. Interestingly Syde2 also contains degradation signals with reflecting the relatively lower Syde2 expression than Syde1 in the developing brain (www.brainspan.org). Fbw7 is one of the listed SCF-E3 ligases involved in neuronal differentiation^78,79^ and the regulation of astrocyte generation, and proteasome-mediated degradation of Syde2 may be involved in its regulation of RhoGTPase during neurodevelopment.

## Supplementary Information

Supplementary Information.
