# Supplementary material for: Function of SYDE C2-RhoGAP family as signaling hubs for neuronal development deduced by computational analysis
Source: Sci Rep. 2022 Mar 12;12:4325. doi: 10.1038/s41598-022-08147-7 (PMC8918327; doi:10.1038/s41598-022-08147-7)
Supplement: Supplementary file 1 — Supplementary Information. [file 41598_2022_8147_MOESM1_ESM.pdf]

Figure S1

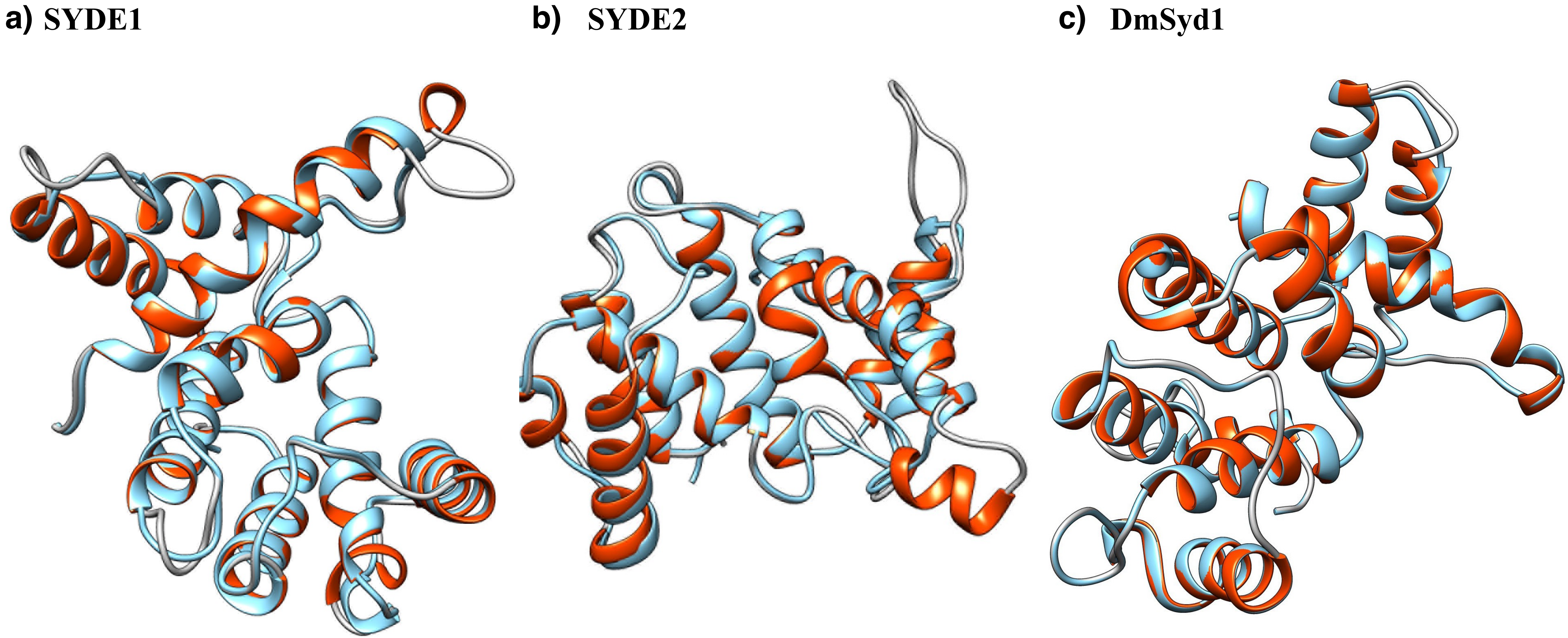

Figure S1. Superposition of SYDE RhoGAP domains

SYDE RhoGAP domain, colored according to secondary structure (red: alpha-helices; gray: loops) is superimposed to template (5C2K: MgcRacGAP, 3CXL: ArhGAP2 in light blue). **a**: HsSYDE1 and 5C2K superposition. **b**: HsSYDE2 and 5C2K superposition. **c**: DmSyd1 and 3CXL superposition. In each case, the SYDE RhoGAP domain signature is superimposed to template catalytic domain by UCSF Chimera software. The structures were visualized using UCSF Chimera software version 1.15 (http://www.cgl.ucsf.edu/chimera).

Figure S2

a) SYDE1 **b) SYDE2**

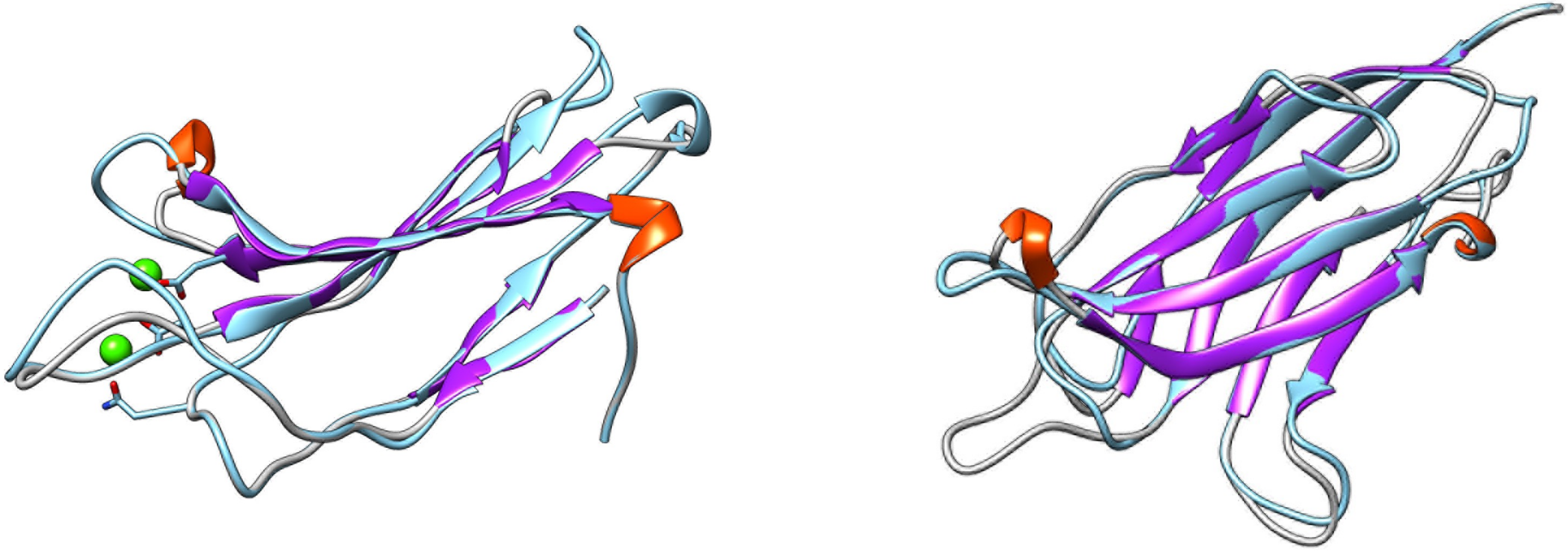

Figure S2. Superposition of SYDE C2 domains

SYDE C2 domain, colored according to secondary structure (red: alpha-helices; purple: beta-sheets; gray: loops) is superimposed to template (1DJI: PLCdelta1, 5IZ5: GIVD-cPLA$_2$, in light blue). **a**: HsSYDE1 and 1DJI superposition. **b**: HsSYDE2 and 5IZ5 superposition. In both cases, the SYDE RhoGAP domain signature is superimposed to template domain by Chimera software.

Table S1

Tools	URL	Ref.
BioGrid	https://thebiogrid.org/	38
D2P2	https://d2p2.pro/	22
ELM	http://elm.eu.org/	21
ERRAT	https://saves.mbi.ucla.edu/	a)
HHPred	https://toolkit.tuebingen.mpg.de/tools/hhpred	30
IntAct	https://mobidb.bio.unipd.it/	39
InterPro	https://www.ebi.ac.uk/interpro/	18
I-Tasser	http://zhanglab.ccmb.med.umich.edu/I-TASSER	32
KEGG	https://www.genome.jp/kegg/	40
MAFFT	https://mafft.cbrc.jp/alignment/server/large.html	16
MEGA11	https://www.megasoftware.net/	29
MobiDB	https://mobidb.bio.unipd.it/	20
Procheck	https://saves.mbi.ucla.edu/	b)
PSIPRED	http://bioinf.cs.ucl.ac.uk/psipred/	19
SwarmDock	https://bmm.crick.ac.uk/~svc-bmm-swarmdock/	36
SwissPalm	https://swisspalm.org/	27
SwissModel	https://swissmodel.expasy.org/	34
trRosetta	https://yanglab.nankai.edu.cn/trRosetta/	33

Table S1 Tools used for in silico analysis of SYDE proteins.

Ref. indicate the literatures listed in References except a) Colovos, C. & Yeates, T. O. Verification of protein structures: Patterns of nonbonded atomic interactions. *Protein Sci.* **2**, 1511-1519 (1993) and b) Wlodawer, A. Stereochemistry and validation of macromolecular structures. *Methods. Mol. Biol.* **1607**, 595-610 (2017)

Table S2

Target	Template	Prob	E-value	Score	SS	Cols	Query HMM	Template HMM	Id(%)	Qmean
HsSYDE1 RhoGAP	5c2k	99.90	3.40E-21	140.2	21.1	177	11-205	357-537(415)	31.7	0.66
HsSYDE1 C2	1dji	99.01	9.50E-9	72.72	11.0	97	1-92	631-729(624)	17.6	0.55
HsSYDE2 RhoGAP	5c2k	99.85	3.40E-19	135.46	19.2	175	2-214	350-537(415)	32.4	0.64
HsSYDE2 C2	5iz5	98.51	5.20E-5	46.51	13.7	108	14-131	19-138(814)	15.7	0.48
DmSyd1 RhoGAP	3cxl	99.88	2.50E-21	141.42	15.8	189	1-199	266-455(463)	31.6	0.74

Table S2. HHpred template search summary. Details of best hits for the best template of SYDE-family proteins are indicated. **Prob**: Probability of template to be a true positive. **E-value**: expect-value (average hit of false positives) **Score**: raw score calculated by comparing the amino acid distributions between columns from the query alignment and columns from the template alignment. The probabilities for insertions and deletion at each position in the alignment are taken into account as positional specific gap penalties. **SS**: secondary structure score**. Cols**: The number of aligned match-match column in the HMM-HMM alignment. **Query HMM**: Range of query match states aligned. **Template HMM**: range of template match states aligned and total number of template HMM as indicated in parenthesis. **Id(%)**: sequence identity. **Qmean**: global score of the whole model reflecting the predicted reliability for modeling which ranges from 0 to 1. **5c2k.pdb**: Structure of MgcRacGAP bound to RhoA **1dji.pdb**: Structure of C2 domain of Phospholipase Cδ1 complex with Ca^{2+}. **5iz5.pdb**: Structure of GIVD cytosolic phospholipase A2. **3cxl.pdb**: Structure of chimerin1.
